# *In vivo* proximity-labeling with *mini*TurboID to screen for protein-protein interactions in the filamentous ascomycete *Sordaria macrospora*

**DOI:** 10.1016/j.mex.2025.103351

**Published:** 2025-05-07

**Authors:** Lucas S. Hollstein, Svenja Groth, Kerstin Schmitt, Oliver Valerius, Stefanie Pöggeler

**Affiliations:** a Department of Genetics of Eukaryotic Microorganisms, Institute of Microbiology and Genetics, Georg-August-University of Göttingen, Grisebachstr. 8, 37077 Göttingen, Germany; b Department of Molecular Microbiology and Genetics, Institute of Microbiology and Genetics, Georg-August-University of Göttingen, Grisebachstr. 8, 37077 Göttingen, Germany; c Service Unit LCMS Protein Analytics, Göttingen Center for Molecular Biosciences (GZMB), Georg-August-University of Göttingen, Grisebachstr. 8, 37077 Göttingen, Germany

**Keywords:** BioID, Filamentous fungi, TurboID, MiniTurbo, MiniTurboID, Protein-protein interactions, Proximity-dependent labeling, Proximity-labeling with miniTurboID to screen for protein-protein interactions

## Abstract

The Biotin Identification (BioID) method applies proximity-dependent labeling of co-localizing proteins to screen for protein-protein interactions *in vivo*. Therefore, the protein of interest (POI) is fused to a promiscuous biotin ligase. This ligase covalently biotinylates proximal proteins, which allows their specific enrichment and subsequent identification by mass spectrometry. In recent research, the BioID method was applied in the filamentous ascomycete *Sordaria macrospora* using a codon optimized TurboID ligase. In this study, we applied a smaller variant of the TurboID biotin ligase, named *mini*TurboID to perform BioID experiments in *S. macrospora*. Here, we provide a comprehensive and detailed guideline of experimental steps for the application of BioID in filamentous fungi.

• The BioID method screens for protein-protein interactions via *in vivo* labeling of nearby proteins through a biotin ligase, which is fused to the POI
• TurboID and *mini*TurboID ligases can be used for BioID experiments in the filamentous fungus *Sordaria macrospora*

## Graphical abstract

**Figure:**
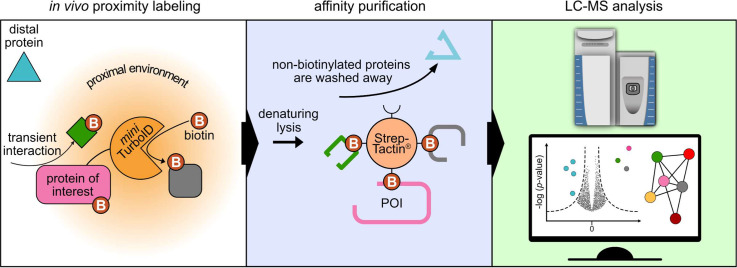

Specifications table

**Table:** 

Subject area:	Biochemistry, Genetics and Molecular Biology
More specific subject area:	Protein-protein interactions
Name of your method:	Proximity-labeling with miniTurboID to screen for protein-protein interactions
Name and reference of original method:	Hollstein, L. S., Schmitt, K., Valerius, O., Stahlhut, G., & Pöggeler, S. (2022). Establishment of *in vivo* proximity labeling with biotin using TurboID in the filamentous fungus *Sordaria macrospora. Scientific reports, 12*(1), 17,727. https://doi.org/10.1038/s41598–022–22545-x
Resource availability:	Plasmids are available on request. LC-MS data has been submitted to the ProteomeXchange Consortium via the PRIDE partner repository with the dataset identifier PXD061752.

## Background

Biotin Identification (BioID) is a powerful method that employs an *in vivo* labeling strategy to screen for potential protein-protein interactions in cells of animals, plants, yeasts and filamentous fungi [[1], [2], [3], [4]]. For this method, a biotin ligase is fused to the protein of interest (POI) through genetic engineering. In its native context the *Escherichia coli* BirA ligase converts biotin into the reactive biotinoyl-5′-AMP intermediate and covalently links it to specific lysine residues of its target protein. However, this system can be exploited using a missense mutation (R118G) of the *BirA* gene (BirA*) [1]. This mutation reduces the affinity for biotinoyl-5′-AMP, thus resulting in a premature and promiscuous release of the reactive intermediate [5]. Once released from the ligase, biotinoyl-5′-AMP diffuses and covalently attaches itself to primary amines (e.g. lysine) of nearby proteins in a non-specific manner. In contrast, natural biotinylation of proteins is a rare and highly specific post-translational modification. Biotinylated proteins can be selectively enriched using streptavidin or derivates of it. Combination of proximity-dependent biotin labeling with liquid chromatography coupled to mass spectrometry (LC-MS) allows unbiased screening for proteins that co-localize within the same cellular microenvironment as the POI, indicating a putative protein-protein interaction. As nearby proteins are labeled in a cumulative and covalent manner, the cell lysis can be carried out in the presence of denaturing reagents. Hence, BioID supports the identification of membrane-associated or insoluble proteins, including weak and transient protein-protein interactions.

Several biotin ligases have been engineered for biotin-driven proximity labeling [[6], [7], [8]]. Among them are two promiscuous BirA variants, called TurboID and *mini*Turbo [7]. For better readability, the *mini* of *mini*Turbo is written in italics here. Both enzymes exhibit accelerated labeling kinetics, and a shifted temperature profile compared to BirA*. The *mini*Turbo ligase is approximately 7 kDa smaller than TurboID and might therefore be less likely to perturb the function or subcellular targeting of the POI it is fused to. In recent research, we applied the BioID method for the filamentous ascomycete *Sordaria macrospora* (Sm) [4]. We fused a Sm codon-optimized TurboID biotin ligase to a component of the striatin-interacting phosphatase and kinase (STRIPAK) complex for proof of concept. This multiprotein complex regulates developmental processes such as hyphal fusion, fruiting body development and vegetative growth in *S. macrospora* [9,10]. With the TurboID ligase fused to the STRIPAK complex interactor 1 (SCI1), we significantly enriched the already known STRIPAK subunits PRO11, PRO22, SmMOB3 and SmPP2Ac1, thus demonstrating the successful application of TurboID. To test whether the smaller *mini*Turbo ligase can be applied for *S. macrospora*, we cloned a construct expressing a shortened TurboID and named it *mini*TurboID. The expression of the SCI1-*mini*TurboID fusion protein in *S. macrospora* was verified by Western blot, and a BioID experiment was performed. In this experiment proteins significantly enriched in the SCI1-TurboID experiments were identified with SCI1-*mini*TurboID, with comparable intensities and spectral counts. Thus, we conclude that *mini*TurboID is functional in *S. macrospora*. Here, we provide a step by step guide for the BioID experiment including LC-MS sample preparation.

## Method details

### Buffers

1. Lysis buffer: 10 mM Tris pH 7.5, 150 mM NaCl, 0.5 mM EDTA pH 8, 1 mM PMSF, 2 mM DTT, 0.5 % Nonidet-P40, 2x c0mplete™ EDTA-free Proteinase Inhibitor Cocktail, 1x PhosSTOP™, 4 % SDS
2. 10x Buffer W: 1 M Tris-Cl, 1.5 M NaCl, 10 mM EDTA, pH 8
3. 10x Buffer BXT: 1 M Tris-Cl, 1.5 M NaCl, 10 mM EDTA, 500 mM biotin, pH 8
4. SDS-PAGE running buffer: 250 mM Tris base, 1.92 M glycine, 35 mM SDS
5. Fixing solution: 10 % acetic acid (glacial), 40 % EtOH
6. Trypsin digestion buffer: 20 ng/µL trypsin in 1 mM acetic acid, 24.5 mM NH_4_HCO_3_
7. LC-MS sample buffer: 2 % acetonitrile, 0.1 % formic acid

### Reagents

acetonitrile ammonium hydrogencarbonate (NH_4_HCO_3_) ammonium Persulfate (APS) chloroform cOmplete™ EDTA-free Proteinase Inhibitor Cocktail (Roche 04693132001) dithiothreitol (DTT)

Empore™ C18 Extraction Disks (3 M, 2215) ethanol ethylenediaminetetraacetic acid (EDTA) formic acid iodoacetamide methanol monoclonal Anti-HA antibody produced in mouse -clone HA-7- (Sigma-Aldrich Co, H9658)

N,N,N′,N′ -Tetramethylethylenediamine (TEMED)

PageRuler™ prestained protein ladder (Thermo Scientific™, 26616) phenylmethylsulfonyl fluoride (PMSF)

PhosSTOP™ (Roche, 04906837001) polyacrylamide gel for SDS-PAGE

• 5 % stacking gel: 12.5 % Rotiphorese^Ⓡ^ Gel40, 0.125 M Tris–HCl pH 6.8, 0.11 % SDS, 0.09% TEMED, 0.067 % APS
• 12 % resolving gel: 30 % Rotiphorese^Ⓡ^ Gel40, 0.375 M Tris–HCl pH 8, 0.11 % SDS, 0.0916 % TEMED, 0.08 % APS
  Ponceau S solution: 0.1 % Ponceau S (Sigma Aldrich, P3504), 5 % glacial acetic acid
  Protein LoBind^Ⓡ^ tubes 1.5 mL (Eppendorf SE, 022431081)
  Rotiphorese^Ⓡ^ Gel40 (Roth, T802.1)
  SDS sample buffer (Serva, 42527.01)
  sodium dodecyl sulphate (SDS)
  Strep-Tactin^Ⓡ^ Sepharose^Ⓡ^ resin (IBA Lifesciences GmbH, 2–1201–002)
  • Note: Streptavidin is a homotetrameric protein from the bacterium *Streptomyces avidinii*, which is known for its extraordinarily high affinity and specificity for biotin. While this high affinity is favorable for the enrichment of biotinylated proteins from cell lysates, it poses challenges for the elution of the biotinylated proteins for downstream processing. To avoid harsh elution conditions, we use an engineered variant of streptavidin, called Strep-Tactin^Ⓡ^ (IBA Lifesciences GmbH) for the affinity purification of biotinylated proteins in BioID experiments. Strep-Tactin^Ⓡ^ was created by site-directed mutagenesis of streptavidin’s flexible loop that covers the binding site for biotin. This reduced the binding affinity for biotin, allowing reversible binding and mild competitive elution of biotinylated proteins from Strep-Tactin^Ⓡ^ by addition of biotin [11].
  trypsin sequencing grade (Serva, 37283.01)

### General remarks for preparation of LC-MS samples

• Always wear fresh gloves (vinyl gloves or powder free nitrile gloves)
• Try to avoid plastics, never store solvents in plastic vials and avoid glassware that was cleaned in a dishwasher

### Strain construction

**Fig. 1:**
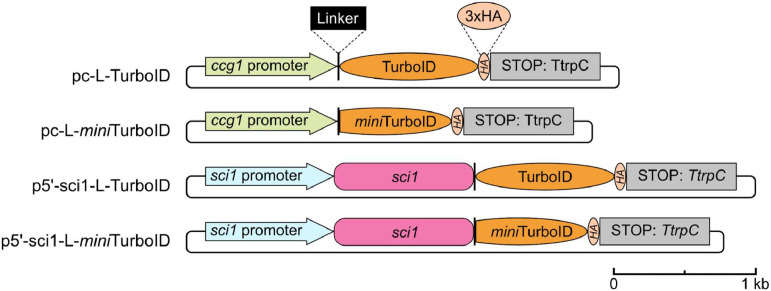
Schematic illustration of the plasmids used for strain construction for BioID experiments. The control constructs pc-L-TurboID and pc-L-*mini*TurboID express a free, unfused TurboID or *mini*TurboID biotin ligase under control of the constitutive promoter of the *clock-controlled gene 1* (pc) from *Neurospora crassa*. SCI1 constructs consist of the *sci1*-ORF fused to either TurboID or *mini*TurboID. Here, expression is controlled by the native *sci1* promoter (p5’). TurboID constructs encode a MGGGGSGGGGS linker, while *mini*TurboID constructs encode a MGGGGSGGGSGGGGS linker at the N-terminus of the biotin ligase. A 3x HA tag (3x YPYDVPDYA) is fused to the C-terminus of the biotin ligases. Expression is terminated by the terminator of the *anthranilate synthase* gene from *Aspergillus nidulans* (TtrpC); a scalebar is indicated.

**Fig. 2:**
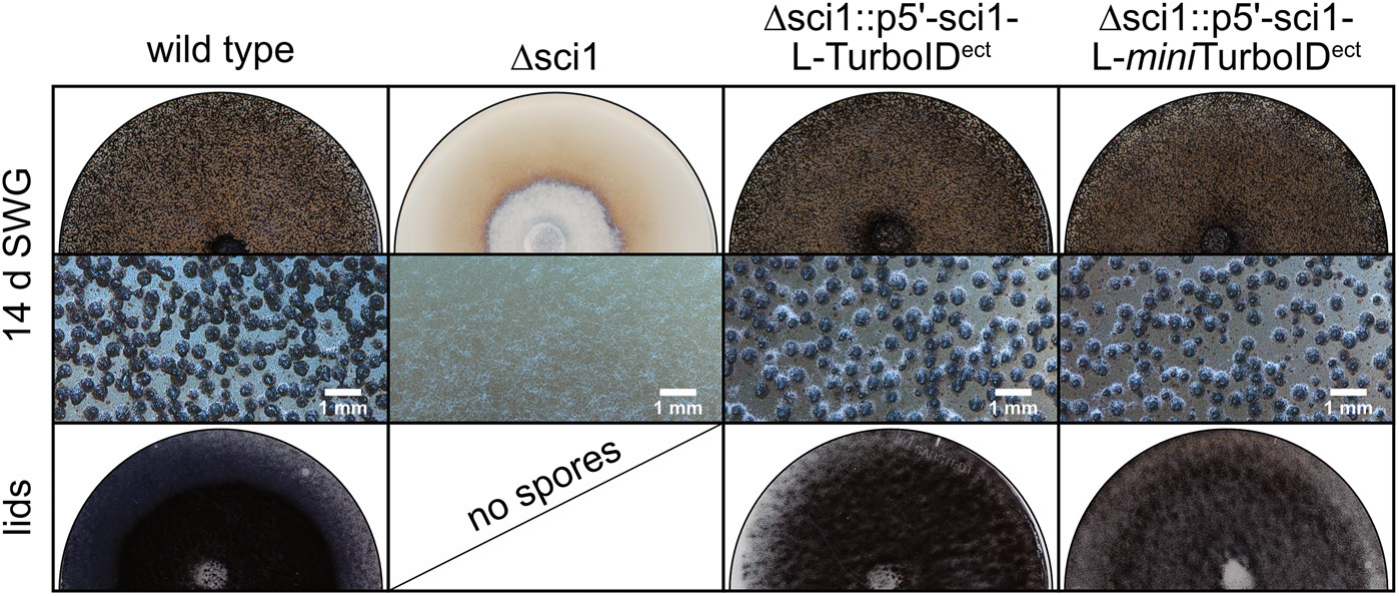
Complementation of the sterile Δsci1 strain by ectopic integration of sci1-(*mini*)TurboID constructs. The single spore isolates were grown at 27 °C on solid *S**ordaria*Westergaards (SWG) medium. The plates and the spore-covered lids were documented after 14 days. Expression of the sci1-(*mini*)TurboID fusion constructs is regulated by the native *sci1* promoter (p5’). Inlays show a magnification of the plate, a scale bar is indicated. ect, ectopically integrated.

To allow optimal expression of the recombinant protein, the TurboID sequence has been codon optimized for expression in *S. macrospora*. During cloning, the sequence of L-*mini*TurboID including the C-terminal 3x HA-tag was amplified from the plasmid pc-L-TurboID [4], thus incorporating the TurboID-specific mutations M241T and S263P into the *mini*TurboID construct (Fig. 1, Supplementary Table S1–2 and Figure S1). Since the thereby created construct more closely resembles a shortened TurboID, rather than the *mini*Turbo ligase developed by Branon and colleagues [7], we named it *mini*TurboID. The *sci1* promoter and *sci1*open reading frame (ORF) were amplified from the plasmid p5’-sci1-L-TurboID [4]. Plasmid construction was carried out in the *Saccharomyces cerevisiae* strain PJ69–4A via homologous recombination [12]. The assembled plasmid p5’-sci1-L-*mini*TurboID was transformed into the sterile *S. macrospora* Δsci1 deletion strain [9] via ectopic integration and complemented the deletion strain phenotype (Fig. 2). The plasmids pc-L-TurboID and pc-L-*mini*TurboID were transformed into the *S. macrospora* wild type. For wt::pc-L-TurboID, single spore isolates were generated and expression was verified by Western blot detection of the 3x HA tag [4]. Transformations of the wild type with the plasmid pc-L-*mini*TurboID yielded fertile heterokaryotic primary transformants, but no homokaryotic single spore isolates could be isolated. Western blot analysis of the heterokaryotic primary transformants displayed very low expression levels of *mini*TurboID in comparison to TurboID (data not shown). In literature, *mini*Turbo has been reported to exhibit cytotoxic effects [7,13]. Therefore, we did not pursue the transformation of pc-L-*mini*TurboID any further. It is possible that the expression of free *mini*TurboID in *S. macrospora* is achievable using promoters with lower expression levels.

### Sampling

For the BioID experiments, three biological replicates of each *S. macrospora* strain expressing either the free TurboID control, the SCI1-TurboID or SCI1-*mini*TurboID fusions were grown for 3 days in liquid Biomalt maize medium (BMM) under continuous light conditions at 27 °C. After three days, the cultures were supplemented with 10 µM biotin for either 30 min (free TurboID), or 4 h (SCI1-TurboID and SCI1-*mini*TurboID). The mycelium was pressed dry to remove residual medium, frozen in liquid N_2_ and stored at −80 °C. Proteins were extracted from the frozen mycelium as described below.

### Protein extraction from frozen mycelium

1. Grind mycelium in liquid N_2_ and transfer mycelium powder to falcon tube
  a. Add 500 µL Lysis buffer per 1 g mycelium powder, vortex to mix
2. Heat proteins for 5 min at 65 °C
3. Centrifuge 10,000 x g for 20 min at room temperature (RT)
4. Transfer crude protein extract (supernatant) to a fresh microcentrifuge tube

### Biotin affinity purification using Strep-Tactin^Ⓡ^ Sepharose^Ⓡ^

1. Equilibration of the Strep-Tactin^Ⓡ^ Sepharose^Ⓡ^ resin at RT
  a. Pipette 100 µL Strep-Tactin^Ⓡ^ Sepharose^Ⓡ^ slurry (50 µL bed volume)
  b. Centrifuge 2 min at 400 x g to sediment the resin
  c. Remove storage buffer and wash twice with 375 µL 1x Buffer W
2. Capture of biotinylated proteins and washing
  a. Remove Buffer W, add 1 ml of crude protein extract (equals to roughly 30 mg protein) and incubate for 30 min on a rotating wheel
  b. Centrifuge 400 x g, 2 min and take off unbound fraction
  c. Wash the resin 3 times with 800 µL 1x Buffer W with 0.4 % SDS (invert 5 times to mix)
3. Elution of biotinylated proteins with Buffer BXT
  a. Add 100 µL of 1x BXT to the resin and incubate for 10 min on a rotating wheel
    i. Ensure that the BXT is in contact with the resin
  b. Centrifuge and collect the eluate (supernatant) in a Protein LoBind^Ⓡ^ tube, repeat the elution for a total of 200 µL eluate
  c. Split the eluate equally into 2 Protein LoBind^Ⓡ^ tubes and perform the chloroform-methanol precipitation [14] with 100 µL of eluate per tube. This prevents excessive volumes during chloroform-methanol precipitation.
4. Chloroform-methanol precipitation of biotinylated proteins
  a. Work on ice, use cooled solutions and centrifuge at 4 °C
  b. Each tube should contain 100 µL of eluate
  c. Add 400 µl methanol to the sample, vortex 5 sec to mix
  d. Add 100 µl chloroform, vortex 5 sec to mix
  e. Add 300 µl water, vortex the sample 15 sec to mix, then centrifuge the sample at 4000 x g for 3 min. The sample separates into 2 phases: a large aqueous layer on top and a smaller chloroform layer at the bottom.
  f. Discard the upper phase: do not destroy the interphase, which contains the protein. It is advisable to leave 10–20 µl of the upper phase.
  g. Add 300 µl methanol, vortex to mix, then centrifuge at 20,000 x g for 10 min at 4 °C The protein will sediment forming a white pellet.
  h. Remove supernatant and dry the sample in the SpeedVac concentrator at 60 °C
  i. Store the protein pellet at −20 °C.

### SDS-polyacrylamide gel electrophoresis (PAGE)

1. Resuspend the protein pellets in 1x SDS sample buffer and incubate for 5 min at 85 °C.
2. Reunite the protein pellets that were split for chloroform-methanol precipitation, so that each lane of the polyacrylamide gel is loaded with one biological replicate. Separate each lane with a protein ladder serving as a spacer to avoid contamination among the samples.
3. Let the gels run shortly until the dye front has moved ∼2,5–3 cm into the resolving gel.

### In-gel digestion based on Shevchenko et al. (1996) [15]

*Volumes can vary; the volume of buffer must be adjusted so that the gel pieces are covered with the solution.

1. Incubate each gel in 50 ml fixing solution for at least 45 min (the gel shrinks)
2. Incubate gels in H_2_O until they resize
3. Cut each lane into 2 to 3 fractions using a clean scalpel
  a. Gel pieces should be no smaller than 2 mm x 2 mm
  b. Gel pieces can be stored in Protein LoBind^Ⓡ^ tubes at 4 °C
  c. Do not cut the gel pieces too small, they might clog the pipettes in later steps
4. Add 30 µl* acetonitrile to the gel pieces, shake in a thermomixer for 10 min at RT
  a. The gel pieces will become opaque and shrink
5. Remove acetonitrile, dry the gel pieces for 10 min in the SpeedVac concentrator at 60 °C
6. Add 150 µl 10 mM DTT (in 100 mM NH_4_HCO_3_), incubate for 1 h at 56 °C
7. Spin down condensation and take off DTT solution
8. Add 150 µl 55 mM iodoacetamide (in 100 mM NH_4_HCO_3_) to the pieces, incubate for 45 min at RT in the dark
9. Remove iodoacetamide and wash pieces with 150 µl 100 mM NH_4_HCO_3_, shake 10 min at RT, discard the solution
10. Add 150 µl acetonitrile, shake 10 min at RT, take off acetonitrile
11. Repeat the washing with 100 mM NH_4_HCO_3_ and acetonitrile one more time
12. Dry the gel pieces for 10 min in the SpeedVac concentrator at 60 °C
13. Add 30 µl* trypsin digestion buffer (20 ng/µL) to the gel pieces and incubate for 45 min on ice
14. Discard residual trypsin digestion buffer that was not taken up by the gel pieces
15. Add 30 µl* 25 mM NH_4_HCO_3_ pH 8.0 and incubate over night at 37 °C

From now on all supernatants, containing the peptides, are collected. Fractions belonging to one biological replicate are pooled during peptide extraction.

1. Collect the supernatants in Protein LoBind^Ⓡ^ tubes
2. Add 30 µl* 20 mM NH_4_HCO_3_ to the gel pieces, shake for 10 min at RT
3. Add 30 µl* 50 % acetonitrile, 5 % formic acid to the gel pieces, shake for 20 min at RT
4. Repeat the extraction with 50 % acetonitrile, 5 % formic acid twice
5. Dry the supernatants completely in the SpeedVac concentrator at 60 °C
6. Peptide pellets can be stored at −20 °C

### C18 StageTip desalting based on Rappsilber et al. (2003) [16]

1. Prepare StageTips with at least one layer of C18 material
  a. The assembly of a StageTip is described in Rappsilber et al. (2007) [17].
  b. StageTips can be placed in standard microcentrifuge tubes, which have a hole cut into their lids. It is advisable to prepare cut-off lids to serve as reusable adapters (Supplementary Figure S2).
2. Resuspend each peptide pellet in 20 µL LC-MS sample buffer
  a. Mix by pipetting and shaking in a thermomixer
  b. Optionally: incubate for 3 min in an ultrasonic bath
3. Conditioning of StageTips
  a. Wash StageTips with 100 µL methanol, 0.1 % formic acid and spin down for 2 min at 1000 x g, discard the flow through
  b. Wash with 100 µL 70 % acetonitrile, 0.1 % formic acid, spin down
  c. Wash with 100 µL 0.1 % formic acid, spin down
  d. Repeat washing with 100 µL 0.1 % formic acid once
4. Loading
  a. Put StageTips into fresh microcentrifuge tubes to gather the flow through for reloading
  b. Load the peptides (in 20 µL LC-MS sample buffer) onto the StageTips
  c. centrifuge 2 min 1000 x g
  d. Reload flow through and centrifuge again
5. Washing
  a. Wash StageTips twice with 180 µL of 0.1 % formic acid
6. Elution
  a. Transfer the StageTips into fresh 1.5 ml Protein LoBind^Ⓡ^ tubes
  b. Add 60 µL 70 % acetonitrile, 0.1 % formic acid
  c. Incubate 1 min and centrifuge 5 min 1500 x g
  d. Dry the eluates completely in the SpeedVac at 60 °C
  e. Store peptide pellet at −20 °C

## Method validation

### LC-MS analysis

Processed BioID samples were analyzed with LC-MS by the Service Unit LCMS Protein Analytics of the Göttingen Center for Molecular Biosciences (GZMB) of the Georg-August University of Göttingen.

Dried peptide samples were reconstituted in 20 µl LC-MS sample buffer (2 % acetonitrile, 0.1 % formic acid). 2 to 8 µl of each sample were subjected to reverse phase liquid chromatography for peptide separation using an RSLCnano Ultimate 3000 system (Thermo Fisher Scientific): Peptides were loaded on an Acclaim PepMap 100 pre-column (100 µm x 2 cm, C18, 5 µm, 100 Å; Thermo Fisher Scientific) with 0.07 % trifluoroacetic acid at a flow rate of 20 µL/min for 3 min. Analytical separation of peptides was done on an Acclaim PepMap RSLC column (75 µm x 50 cm, C18, 2 µm, 100 Å; Thermo Fisher Scientific) at a flow rate of 300 nL/min. The solvent composition was gradually changed within 94 min from 96 % solvent A (0.1 % formic acid) and 4 % solvent B (80 % acetonitrile, 0.1 % formic acid) to 10 % solvent B within 2 min, to 30 % solvent B within the next 58 min, to 45 % solvent B within the following 22 min, and to 90 % solvent B within the last 12 min of the gradient. All solvents and acids had Optima grade for LC-MS (Fisher Chemical). Eluting peptides were on-line ionized by nano-electrospray (nESI) using the Nanospray Flex Ion Source (Thermo Fisher Scientific) at 1.5 kV (liquid junction) and transferred into a Q Exactive HF mass spectrometer (Thermo Fisher Scientific). Full scans in a mass range of 300 to 1650 *m/z* were recorded at a resolution of 30,000 followed by data-dependent top 10 HCD fragmentation at a resolution of 15,000 (dynamic exclusion enabled). LC-MS method programming and data acquisition was performed with the XCalibur 4.0 software (Thermo Fisher Scientific).

The LC-MS raw files were analyzed using MaxQuant (v1.6.10.43) configured with standard parameters and the following changes:

• Modifications: add “Biotinylation of Lysine” (Unimod accession #3) as variable modification
• Optional: add “Phospho (STY)” as variable modification
• Label-free quantification: activate LFQ
• Digestion, max missed cleavage sites: 3

The proteinGroups.txt output file from MaxQuant was imported into Perseus (v1.6.15.0) for statistical analysis as described in Table 1.

**Table 1:** Workflow for statistical analysis of the MaxQuant output with Perseus (v1.6.15.0).

**#**	**Command**	**Description**
1	Generic matrix upload	proteinGroups.txt Main: LFQ intensities Numerical: MS/MS counts Multi-numerical: Biotin site positions, Phospo(STY) site positions
2	Filter rows based on categorical column	Remove rows containing “+” for columns “only identified by site”, “reverse” and “potential contaminant”
3	Transform	LFQ intensities: log2(x)
4	Categorical annotation	Create group1 with “free TurboID” and “SCI1-TurboID” subgroups Create group2 with “free TurboID” and “SCI1-*mini*TurboID” subgroups
5	Analysis	Multi scatter plot to check reproducibility of the replicates
6	Filter rows based on valid values	Split the workflow by performing valid value filtering separately for group1 and group2: 3 valid values in at least one group: group1 3 valid values in at least one group: group2 This will result in two separate statistical analyses.
7	Replace missing values from normal distribution	Mode: Separately for each column
8	Volcano plot	Grouping: group1, left “free TurboID”, right “SCI1-TurboID” FDR = 0.01, s0 = 2 Grouping: group2, left “free TurboID”, right “SCI1-*mini*TurboID” FDR = 0.01, s0 = 2 Select significantly enriched proteins on the right side of the volcano plot, export selection (reduce matrix)
9	Imputation	Replace imputed values by NaN

### Phenotypic complementation assay

Transformation of SCI1-(*mini*)TurboID fusion constructs into a *S. macrospora* wild-type strain could result in interference of the native SCI1 with the ectopically introduced SCI1-(*mini*)TurboID fusion protein. Consequently, this might lead to proximity labeling of a non-physiological protein environment. In order to enable native protein levels and physiological function of the (*mini*)TurboID-tagged SCI1 protein, the fusion constructs p5’-sci1-L-TurboID and p5’-sci1-L-*mini*TurboID were transformed into the Δsci1 deletion strain. This transformation yielded complemented transformants: while the Δsci1 deletion strain is sterile and does not produce any fruiting bodies, the complemented strains are fertile and macroscopically indistinguishable from the wild type. Fruiting bodies are formed and eject their ascospores, which cover the lids of the petri dishes (Fig. 2).

### Verification of (*mini*)TurboID expression in *S. macrospora*

**Fig. 3:**
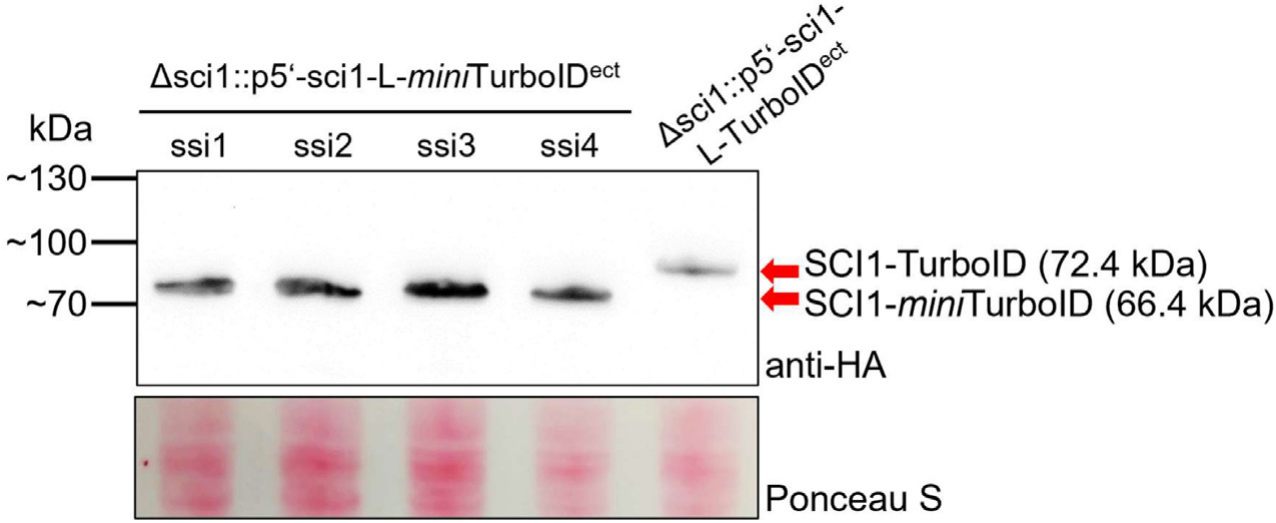
Western blot detection of SCI1-(*mini*)TurboID expression in Δsci1. Expression of both constructs is controlled by the native *sci1* promotor (p5’). Strains were grown in liquid *S**ordaria*Westergaards medium for 4 days at 27 °C. 25 µg of protein were loaded. Ponceau S staining of the membrane served as loading control and Western blotting was performed using a monoclonal anti-HA antibody to detect the C-terminal 3x HA tag of the biotin ligases. The figure shows cropped blots, the full-length membrane images are depicted in Supplementary Figure S3. ect, ectopically integrated; ssi, single spore isolate.

After successful transformation and complementation of the Δsci1 deletion strain, single spore isolates of the strains Δsci1::p5’-sci1-L-TurboID^ect^ and Δsci1::p5’-sci1-L-*mini*TurboID^ect^ were tested for their expression of the HA-tagged biotin ligase fusions via Western blot (Fig. 3). Expression of sci1-TurboID and sci1-*mini*TurboID is controlled by the native *sci1* promoter to ensure authentic expression regulation. This analysis verifies the expression of SCI1-TurboID and SCI1-*mini*TurboID in *S. macrospora*.

### Statistical analysis of the SCI1-BioID LC-MS data

**Fig. 4:**
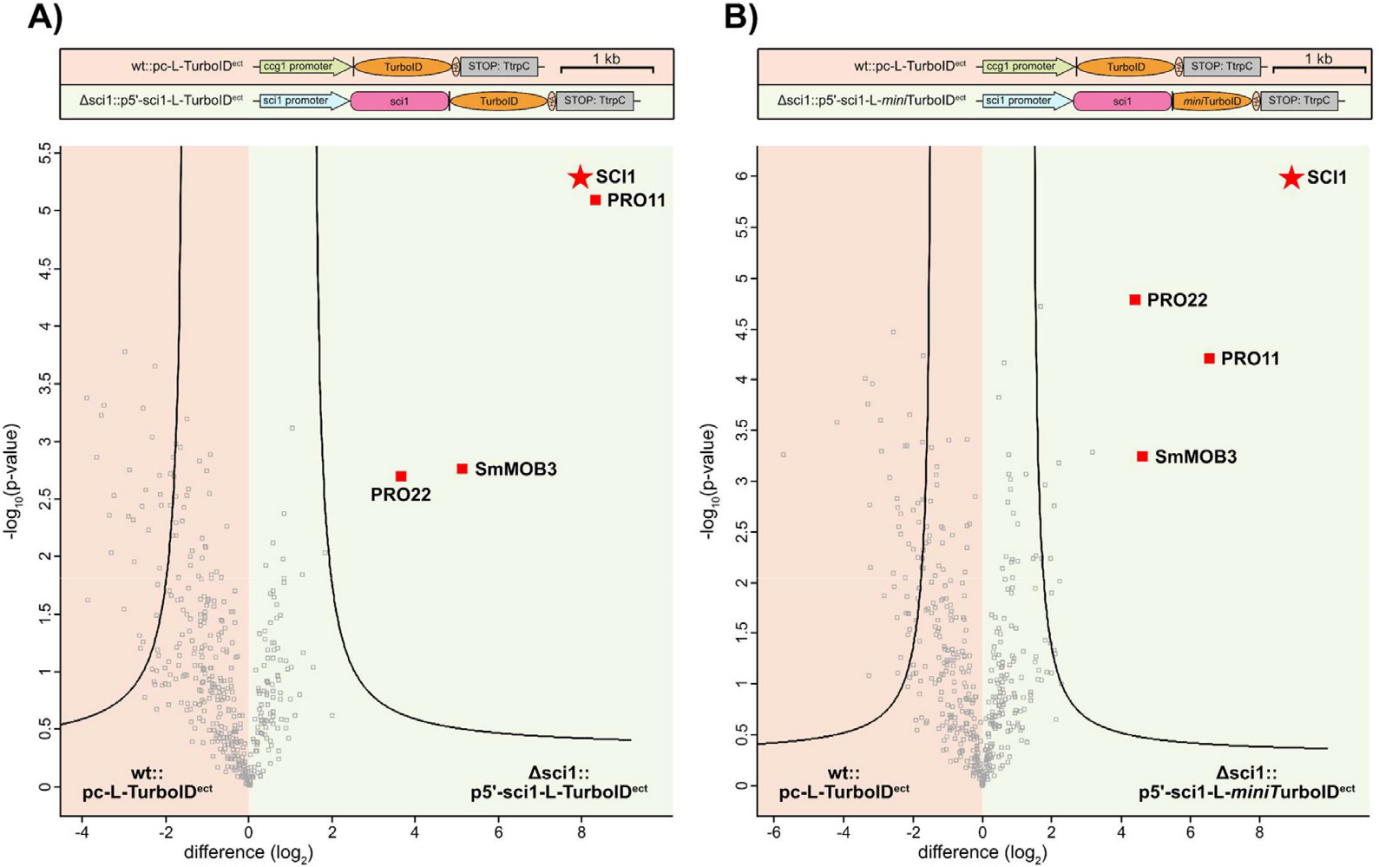
Volcano plot analysis of the SCI1-BioID experiment. The graphs plot the differences in label-free quantification (LFQ) intensities (log_2_ transformed) of the wt::pc-L-TurboID^ect^ control and **A)** the SCI1-TurboID strain Δsci1::p5’-sci1-L-TurboID^ect^ and **B)** the SCI1-*mini*TurboID strain Δsci1::p5’-sci1-L-*mini*TurboID^ect^. The −log_10_(p-value) is plotted on the y-axis. Significantly enriched proteins are separated from non-significant proteins by the plotted curve. The proteins SCI1, PRO11, SmMOB3 and PRO22 are known components of the SmSTRIPAK complex. The POI, SCI1, is marked with a star. Statistical parameters: FDR = 0.01, s0 = 2.

For the SCI1-BioID experiment, 3 biological replicates of the free TurboID (wt::pc-L-TurboID^ect^) control, the SCI1-TurboID (Δsci1::p5’-sci1-L-TurboID^ect^) and SCI1-*mini*TurboID (Δsci1::p5’-sci1-L-*mini*TurboID^ect^) strains were grown. After protein extraction and biotin affinity capture, the proteins were separated by SDS-PAGE and subjected to in-gel digestion using trypsin. The resulting peptides were extracted from the gel and purified by C18 StageTips. The LC-MS raw files were analyzed with MaxQuant (v1.6.10.43) and Perseus (v1.6.15.0) employing the label-free quantification (LFQ) algorithm MaxLFQ [18,19]. The naming scheme of the raw files is listed in Supplementary Table S3. For the statistical analysis, the free TurboID control strain was compared to the SCI1-TurboID strain (Fig. 4A). Since the generation of a *mini*TurboID-specific control strain with plasmid pc-L-*mini*TurboID^ect^ was not successful, the SCI1-*mini*TurboID strain was compared to the full length TurboID control strain in a separate analysis workflow (Fig. 4B). Both statistical analyses show significant enrichment of the POI, SCI1, and the SmSTRIPAK components PRO11, PRO22 and SmMOB3. However, the statistical analysis of SCI1-*mini*TurboID (Fig. 4B) reports more significantly enriched proteins than the SCI1-TurboID analysis (Fig. 4A). Nevertheless, among the significantly enriched proteins in SCI1-*mini*TurboID, the SmSTRIPAK components are the most enriched proteins with the highest statistical confidence and difference in intensity. This indicates that TurboID and *mini*TurboID exhibit different biotinylation profiles, but deliver comparable results in this proof-of-concept experiment.

While the significantly enriched SmSTRIPAK components were identified with high intensities in all SCI1-TurboID and SCI1-*mini*TurboID replicates, they are absent in the free TurboID control. When comparing SCI1-TurboID with SCI1-*mini*TurboID, the proteins SCI1, PRO11, SmMOB3 and PRO22 were quantified with comparable LFQ intensities and similar MS/MS counts (Table 2A+B). Additional support for the functionality of SCI1-*mini*TurboID is demonstrated by the identification of biotinylated peptides (Table 2C). Biotin is a rare post-translational modification, which is mainly restricted to a few protein species, such as carboxylases and histones [20,21]. Therefore, the identification of peptides with biotinylation site information in BioID experiments supports the evidence for *in vivo* proximity with the POI and reduces the risk of false positive results. In this SCI1-BioID experiment, biotin sites have been identified for PRO11, SmMOB3 and the POI. Identification events of biotinylated peptides are evenly distributed among SCI1-TurboID and SCI1-*mini*TurboID (Table 2C). An overview of identified post-translational modifications from the MaxQuant query are shown in Supplementary Figure S4.

**Table 2:** LFQ intensities, MS/MS counts and biotinylation site information of significantly enriched proteins. This table shows parameters of significantly enriched proteins in the SCI1-BioID experiment listed per biological replicate. The proteins SCI1, PRO11, SmMOB3 and PRO22 are known components of the SmSTRIPAK complex. **A)** Protein intensities (log_2_ transformed) after label-free quantification (LFQ). LFQ intensities are color-coded by a gradient: high intensities are shaded in red, while lower intensities are shaded in green. **B)** MS/MS counts **C)** Count of biotinylated lysine sites. NaN, not a number.

The identification of the already known SmSTRIPAK components using the SCI1 subunit with either ligase demonstrates that both, TurboID and *mini*TurboID, can be used to reliably screen for protein-protein interactions. Due to the reduced size of *mini*TurboID (32 kDa), we speculate that it is less likely to interfere with the native function or localization of the POI as compared to the larger TurboID ligase (39 kDa). Furthermore, our *mini*TurboID construct demonstrates that the small ligase is functional when including the TurboID-specific mutations M241T and S263P. Consequently, *mini*TurboID can be easily cloned from existing TurboID vectors in a single PCR amplification reaction. For establishing the BioID proximity labeling methodology in other filamentous fungi, we suggest to test both ligases for their functionality and their compatibility with the POI e.g. by complementation of the knockout with the POI-ligase fusion protein.

In summary, *mini*TurboID extends the molecular toolbox for BioID-based proximity labeling experiments that allows to screen for putative protein-protein interactions. We therefore provide this step by step guide to support other fungal scientists in the establishment of BioID in their own fungal model organism and to serve as a resource for optimization of the BioID methodology.

## Limitations

The BioID methodology has been demonstrated to be a powerful tool for the identification of proteinaceous microenvironments, with the potential to detect protein-protein interactions. However, it is important to consider the inherent limitations of the method:

During the labeling process, the biotin ligase produces the reactive biotinoyl-5′-AMP intermediate that covalently attaches to nearby proteins at primary amines (e.g*.* lysine). Consequently, no labeling is possible if a protein lacks lysine residues or if the lysine residues are not accessible. Lysine residues might be inaccessible because they are shielded by the tertiary structure of the protein itself or by quaternary structure of other proteins in the microenvironment.

Furthermore, the fusion of the biotin ligase to the POI might impair the physiological functionality or localization of the POI’s native context. While this problem applies to every method that relies on tagging of the POI, complementation analyses and reversion of the knockout phenotype to wild-type appearance can help to assess the risks. Additionally, the covalent attachment of biotin at lysine residues of the POI or nearby proteins has the potential to interfere with natural post-translational modifications of lysine such as ubiquitination.

Since the biotinoyl-5′-AMP intermediate disperses from the biotin ligase into the proximity, the identification of a candidate interactor in BioID experiments does not necessarily imply a direct interaction with the POI. Due to the spatial nature of proximity labeling, biotin ligases that have been fused to the N*-* or C-terminus of the POI might face different microenvironments, which might appear contradicting. Thus, it is important to validate the interaction of potential interacting proteins through alternative approaches such as co-immunoprecipitation, yeast-two-hybrid or bimolecular fluorescence complementation experiments.

In order to deal with the background, such as naturally biotinylated proteins or proteins that were enriched non-specifically due to their high abundance in the proteome, appropriate controls are crucial in BioID experiments. In this experiment we utilized a free, unfused TurboID ligase to additionally account for proteins that might bind to the ligase. Other controls can include the side-by-side comparison of two experimental conditions such as drug treatment or stress induction.

## Ethics statements

Not applicable.

## Data availability

The mass spectrometry proteomics data have been deposited to the ProteomeXchange Consortium via the PRIDE partner repository [22] with the dataset identifier PXD061752.

## CRediT authorship contribution statement

**Lucas S. Hollstein:** Project administration, Investigation, Methodology, Visualization, Writing – original draft, Writing – review & editing. **Svenja Groth:** Investigation, Visualization, Writing – original draft, Writing – review & editing. **Kerstin Schmitt:** Investigation, Methodology, Resources, Writing – review & editing. **Oliver Valerius:** Investigation, Methodology, Resources, Writing – review & editing, Funding acquisition. **Stefanie Pöggeler:** Conceptualization, Supervision, Resources, Writing – review & editing, Funding acquisition.

## Declaration of competing interest

The authors declare that they have no known competing financial interests or personal relationships that could have appeared to influence the work reported in this paper.

## Appendix Supplementary materials

Image, application 1
