# Supplementary material for: In vivo proximity-labeling with miniTurboID to screen for protein-protein interactions in the filamentous ascomycete Sordaria macrospora
Source: MethodsX. 2025 May 7;14:103351. doi: 10.1016/j.mex.2025.103351 (PMC12145721; doi:10.1016/j.mex.2025.103351)
Supplement: Supplementary file 1 [file mmc1.pdf]

In vivo proximity-labeling with *mini*TurboID to screen for protein-protein interactions in the filamentous ascomycete *Sordaria macrospora*

Lucas S. Hollstein[1], Svenja Groth[1], Kerstin Schmitt[2,3], Oliver Valerius[2,3], Stefanie Pöggeler[1]*

[1] Department of Genetics of Eukaryotic Microorganisms, Institute of Microbiology and Genetics, Georg-August-University of Göttingen, Grisebachstr. 8, 37077, Göttingen, Germany

[2] Department of Molecular Microbiology and Genetics, Institute of Microbiology and Genetics, Georg-August-University of Göttingen, Grisebachstr. 8, 37077, Göttingen, Germany

[3] Service Unit LCMS Protein Analytics, Göttingen Center for Molecular Biosciences (GZMB), Georg-August-University of Göttingen, Grisebachstr. 8, 37077 Göttingen, Germany

*Correspondence; spoegge@gwdg.de

Table S1: Plasmids used in this study.

Plasmid	Characteristics	Reference
pc-L-TurboID	*pc::L-TurboID::3xHA::TtrpC, hyg^R*	[1]
pc-L-*mini*TurboID	*pc::L-miniTurboID::3xHA::TtrpC, nat^R,* MGGGGSGGGSGGGGS Linker, including TurboID-specific mutations M241T and S263P	This study
p5'-sci1-L-TurboID	*p5'::sci1-L-TurboID::3xHA::TtrpC, nat^R,* MGGGGSGGGGS-Linker	[1]
p5'-sci1-L-*mini*TurboID	*p5'::sci1-L-miniTurboID::3xHA::TtrpC, nat^R,* MGGGGSGGGSGGGGS Linker, including TurboID-specific mutations M241T and S263P	This study

The TurboID sequence was codon optimized according to the codon usage table of *Sordaria macrospora* [2]; *pc*, promoter of the *clock-controlled gene 1* (*ccg1*) from *Neurospora crassa; p5'*, native *sci1* promoter; L, Linker at the N-terminus of (*mini*-)TurboID; *3x HA*, triple HA-tag at the C-terminus of (*mini*-)TurboID; *TtrpC*, terminator of the *anthranilate synthase* gene from *Aspergillus nidulans*; *hyg^R*, hygromycin resistance; *nat^R*, nourseothricin resistance

Table S2: Strains used in this study.

Strain	Characteristics	Reference
wildtype (wt)	wild-type strain, black ascospores, fertile	DSM997
Δsci1	Δsci1::*hyg^R*, black ascospores, sterile, ssi	[3]
wt::pc-L-TurboID^{ect}	ectopic integration of *pc-L-TurboID* into wt, *hyg^R*, fertile, ssi	[1]
Δsci1::p5'-sci1-L-TurboID^{ect}	ectopic integration of *p5'-sci1-L-TurboID* into Δsci1, *hyg^R*, *nat^R*, fertile, ssi	[1]
Δsci1::p5'-sci1-L-*mini*TurboID^{ect}	ectopic integration of *p5'-sci1-L-miniTurboID* into Δsci1, *hyg^R*, *nat^R*, fertile, ssi	This study

ect, ectopically integrated; *pc*, promoter of the *clock-controlled gene 1* (*ccg1*) from *N. crassa; p5'*, native *sci1* promoter; L, Linker at the N-terminus of (*mini*-)TurboID; *3x HA*, triple HA-tag at the C-terminus of (*mini*-)TurboID; *TtrpC*, terminator of the *anthranilate synthase* gene from *A. nidulans*; *hyg^R*, hygromycin resistance; *nat^R*, nourseothricin resistance; ssi, single spore isolates

Table S3: Naming of LC-MS raw files

Raw file	*S. macrospora* strain
**LH89**	wt::pc-L-TurboID^{ect} ssi 1.7
**LH90**	wt::pc-L-TurboID^{ect} ssi 1.8
**LH91**	wt::pc-L-TurboID^{ect} ssi 1.9
**LH92**	Δsci1::p5'-sci1-L-*mini*TurboID^{ect} ssi 1
**LH93**	Δsci1::p5'-sci1-L-*mini*TurboID^{ect} ssi 2
**LH94**	Δsci1::p5'-sci1-L-*mini*TurboID^{ect} ssi 3
**LH95**	Δsci1::p5'-sci1-L-TurboID^{ect} ssi 1.6
**LH96**	Δsci1::p5'-sci1-L-TurboID^{ect} ssi 1.9
**LH97**	Δsci1::p5'-sci1-L-TurboID^{ect} ssi 1.10

ect, ectopically integrated; ssi, single spore isolate

A)

sci1 promoter-*sci1*-ORF(no stop codon)-Linker(MGGGGSGGGSGGGGS)-*mini*TurboID-*3xHA* (including TurboID mutations **M241T** and **S263P**)-*TrpC* terminator

GCTAGCTATCAACATCATCGCATAAACAAATTGTCCATCCAACAGAGCTGCCACTATCCTTCATCCCGACCACA
ACGCAAGACACGGACTCATCGTTACGTCTACATACTTCGTTGTATTAGTAACTCTACCGTCACAATAGTGCTGG
GACGCTCTACGAGTCGCCCTCGATCGGGCTCAGACAAGGACGACGAAGGTCCGGACAAAGACACAGGCGC
ATCCCCAGACTCGCGACAGTCCTTGGAAACCACGACAGGGCAAACAGCCAAATTGGCAAGTAGAGTTCCTCT
GGAGCCTACACCGAATACAAGATCTCCATTCATCCACGGCTCTGTCTTGAATCCACAGCAGCAACGCCGGCC
ACGAGAAGGCTCACTACAGAAGGAGAATAACGACCCTGACCAAGCCCCTTCTTCCAACACACCATCCCGTCG
ACGTAGGGAGATTCTAGAGCCGCCAGCTCTCGAATGGATCCGTGGACAGACACGAACGAGTACATCTAAGGA
CTAGAAACCTGATTCTGCTGCGACTTCGGATCCTTCACCAACACTTCCTTCTGCGCCAAGGCCCCGTTCTGCA
AGCATTGTTCGATCTTCGGACACACCTCGCGCGTCTCGCCTTGACCTAGTTCGAGATATCAGCGGCGATAACG
TCACACAGACCTTGGAAGCTGCCGGTGCTTTTGCTAAGTTTGTTGAGGACGTAAGGAACCTAGAAAAGTCTGT
CACCCGACCTTGTGTACCCAGGTTACCACCCCGGCCGCCTCTCCCTACTCCGCGCAAACTTCCAACATCATC
CAGCGGCCGTCAAAGAGCGAAATCACTCCCAGCCGGCAAAAGAGCCTGGTCGAACCCCAGAAGAGACAAGT
CAGAAGAAACAGACAACGAAGACGAAGAGGACATGAACGGCATGCATGCTGGAAATATGCCCGCGGCCATG
GTTGGCCTCCCTACGCCGGCAGGGCACCAAGCCGAGCTAAATTACATCTACGGCTTGGTTGAGGAGTTGAGC
CGACAGCTTGCGCAGAATCAGCGCGCCCTGGAGGAGGTAGTCTCGGGCGTTGGCAAAGTGCGCGGGCGAG
CAAGGTCACAGTCTCTGACAAATGACGAACTACTCAATGCCGCCGGGGAAGAACTCAAGAGTATGTCTCTCC
CGCCCTCTCATCCTCTCACTTACACGCCCATAACCTTACACTTACTAACTCATCTGTATCTGCATAGACCAAGAC
GAAAACATAGACCAACTAGTCTCCATCCTCACGGAAGCTCTCGAAAAAGCCAAATTCTCTCGCGACGCCAACG
CCGCCCTTTTGTCTCAATACTCGCAAGTCATGTACACCATGCTCAAGAAATTCCACGAGTACAAAGCCAAGCA
CGTGTCCGACGTGGCCGCCTGGCACCACTCGTACCGGGCCCAGCTCGCCGAGGCGCGCGCGGAGAATAGT
CGGCTGCGCGAGCAGATCTGGGAGATGCAGGCGCACGCGGGCAAAGCCAACGAGCTGGTGCGGCGGTTC
CGGGCCGAGTACGACAAGGACGAAAAGCGCTGGGAGCGCAATGTCAATACCAAGGCCGTGAGGCAGGAGC
TGAGGTTCTGGAAACGCATGGCGATGCCGGAACTGCCAGATGATGACGAGTTTTGGAGCGATGATGATGATG
TTATTGATGGGGCGGAGAAGGAGAGGCAGAAGGAGATACAAAAGTTGGATGCGGAGCAGCAGGCGCAGGCT
GCGCAGGCTGTGGCGGAGGAGGCGGCTGCTAATGAGGCTGCTGCTGAAAGTAGTGGTGGGTTGGGAGAGA
TGAGTGGGAGTGGAAGTGGGAGTAATGGTTCTGGCCTGACGGGGGTACCGCCGGAGGTACCACAGCCGCC
GGTTTCCATGGGCGGCGGCGGCAGCGGCGGCGGCAGCGGCGGCGGCGGCTCCATCCCCCTCCTCAACGC
CAAGCAGATCCTCGGCCAGCTCGACGGCGGCTCCGTCGCCGTCCTCCCCGTCGTCGATTCCACCAACCAGT
ACCTCCTCGACCGCATCGGCGAGCTAAAGTCCGGCGACGCCTGCATCGCCGAGTACCAGCAGGCCGGCCG
CGGCTCCCGCGGGCGCAAGTGGTTCTCCCCCTTCGGCGCCAACCTCTACCTCTCCATCTTCTGGCGCCTCA
AGCGCGGCCCCGCCGCCATCGGCCTCGGCCCCGTCATCGGCATCGTCATGGCCGAGGCCCTCCGCAAGCT
CGGCGCCGACAAGGTCCGCGTCAAGTGGCCCAACGACCTCTACCTCCAGGACCGCAAGCTCGCCGGCATC
CTCGTCGAACTCGCCGGCATCACCGGCGACGCCGCCCAGATCGTCATCGGCGCCGGCATCAACGTCGCCAT
GCGCCGCGTCGAGGAGTCCGTCGTCAACCAGGGCTGGATCACCCTCCAGGAAGCCGGCATCAACCTCGAC
CGCAACACCCTCGCCGCC**ACC**CTCATCCGCGAGCTACGCGCCGCCCTCGAACTATTCGAGCAGGAAGGCCT
CGCCCCCTACCTC**CCC**CGCTGGGAGAAGCTCGACAACTTCATCAACCGCCCCGTCAAGCTCATCATCGGCG
ACAAGGAGATATTCGGCATCTCCCGCGGCATCGACAAGCAGGGCGCCCTCCTCCTCGAACAGGACGGCGTC
ATCAAGCCCTGGATGGGCGGCGAGATCAGCCTCCGCTCCGCCGAGAAGAAGCTCGCC*TACCCCTACGACGT*
*CCCCGACTACGCTTACCCATACGATGTGCCCGACTACGCCTACCCATACGATGTGCCAGATTACGCCTAA*GAT
CCACTTAACGTTACTGAAATCATCAAACAGCTTGACGAATCTGGATATAAGATCGTTGGTGTCGATGTCAGCTC
CGGAGTTGAGACAAATGGTGTTCAGGATCTCGATAAGATACGTTCATTTGTCCAAGCAGCAAAGAGTGCCTTC
TAGTGATTTAATAGCTCCATGTCAACAAGAATAAAACGCGTTTTCGGGTTTACCTCTTCCAGATACAGCTCATCT
GCAATGCATTAATGCATTGACTGCAACCTAGTAACGCCTTNCAGGCTCCGGCGAAGAGAAGAATAGCTTAGCA
GAGCTATTTTCATTTTCGGGAGACGAGATCAAGCAGATCAACGGTCGTCAAGAGACCTACGAGACTGAGGAAT
CCGCTCTTGGCTCCACGCGACTATATATTTGTCTCTAATTGTACTTTGACATGCTCCTCTTCTTTACTCTGATAG
CTTGACTATGAAAATTCCGTCACCAGCNCCTGGGTTCGCAAAGATAATTGCATGTTTCTTCCTTGAACTCTCAA
GCCTACAGGACACACATTCATCGTAGGTATAAACCTCGAAATCANTTCCTACTAAGATGGTATACAATAGTAACC
ATGCATGGTTGCCTAGTGAATGCTCCGTAACACCCAATACGCCGGCCGAAACTTTTTTACAACTCTCCTATGAG
TCGTTTACCCAGAATGCACAGGTACACTTGTTTAGAGGTAATCCTTCTTTCTAGAAGTCCTCGTGTACTGTGTA
AGCGCCCACTCCACATCTCCACTCGA

Figure continues next page

B)

sci1-ORF(no stop codon)-Linker(MGGGGSGGGSGGGGS)-*mini*TurboID-*3xHA* (including TurboID mutations **M241T** and **S263P**)

MNGMHAGNMPAAMVGLPTPAGHQAELNYIYGLVEELSRQLAQNQRALEEVVSGVGKVRGRARSQSLTNDELLN
AAGEELKNQDENIDQLVSILTEALEKAKFSRDANAALLSQYSQVMYTMLKKFHEYKAKHVSDVAAWHHSYRAQLA
EARAENSRLREQIWEMQAHAGKANELVRRFRAEYDKDEKRWERNVNTKAVRQELRFWKRMAMPELPDDDEFW
SDDDDVIDGAEKERQKEIQKLDAEQQAQAAQAVAEEAAANEAAAESSGGLGEMSGSGSGSNGSGLTGVPPEVP
QPPVSGGGGSGGGSGGGGSIPLLNAKQILGQLDGGSVAVLPVVDSTNQYLLDRIGELKSGDACIAEYQQAGRGS
RGRKWFSPFGANLYLSIFWRLKRGPAAIGLGPVIGIVMAEALRKLGADKVRVKWPNDLYLQDRKLAGILVELAGITG
DAAQIVIGAGINVAMRRVEESVVNQGWITLQEAGINLDRNTLAA**T**LIRELRAALELFEQEGLAPYL**P**RWEKLDNFIN
RPVKLIIGDKEIFGISRGIDKQGALLLEQDGVIKPWMGGEISLRSAEKKLA*YPYDVPDYAYPYDVPDYAYPYDVPDYA*

Figure S1: Sequences of p5'-sci1-L-*mini*TurboID.

A) Nucleotide sequence **B)** amino acid sequence of p5'-sci1-L-*mini*TurboID. The sequence of the *sci1* open reading frame (ORF) is colored in blue, the linker is colored in yellow and the *mini*TurboID is colored in purple. The DNA sequence of the *sci1* 5' region is colored in grey and the sequence of the terminator of the *anthranilate synthase* gene from *A. nidulans* (*TtrpC)* is colored brown.

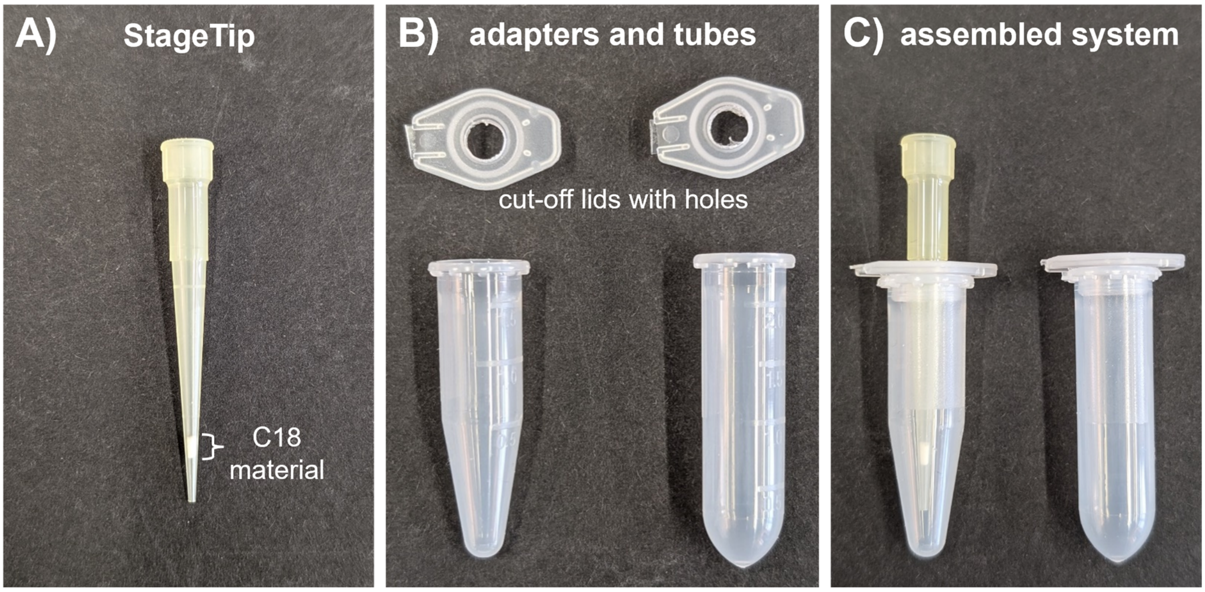

Figure S2: StageTip and reusable adapter system.

StageTips are placed in standard microcentrifuge tubes for conditioning, washing, loading and elution of peptides using reusable adapters. **A)** StageTip consisting of the C18 material placed in a standard 200 µL pipette tip. The StageTip was assembled according to [4] **B)** For the reusable adapters, a hole was drilled into cut-off lids of microcentrifuge tubes (1.5 or 2 ml). **C)** Fully assembled system showing the StageTip placed in a microcentrifuge tube using the adapter. Liquids are passed through the C18 material and collected in the tube by centrifugation.

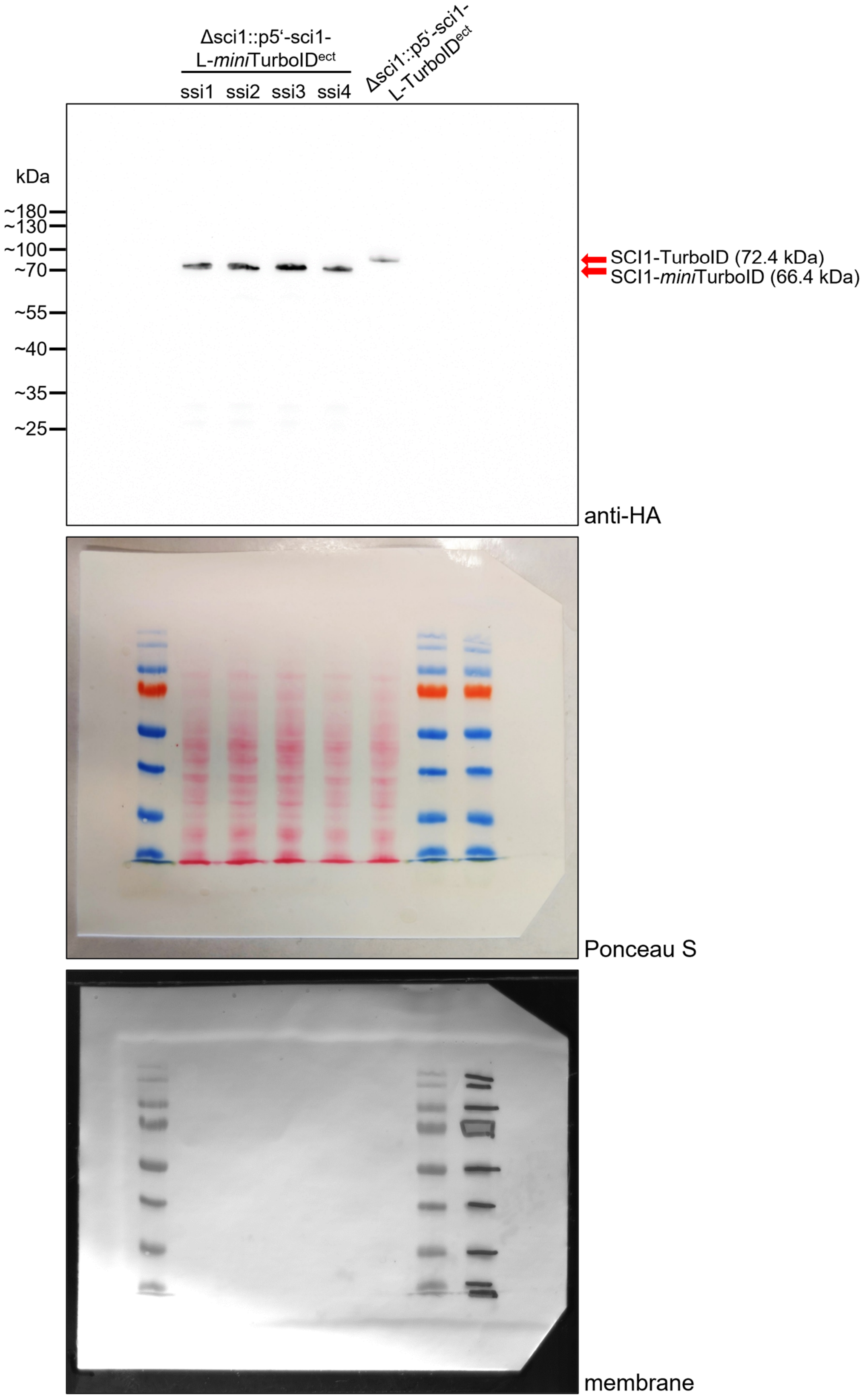

Figure S3: Western blot detection of SCI1-(*mini*)TurboID constructs in Δsci1, full membrane.
Expression of both constructs is controlled by the native *sci1* promotor (p5'). Strains were grown in liquid *Sordaria* Westergaards (SWG) medium for 4 days at 27 °C. 25 µg of protein were loaded. Ponceau S staining of the membrane served as loading control and Western blotting was performed using a monoclonal anti-HA antibody to detect the C-terminal 3xHA tag of (*mini*)TurboID. ect, ectopically integrated; ssi, single spore isolate

Biotinylation of lysines Phosphorylation (STY) Oxidation (M)

>SMAC_05559 SCI1
MNGMHAGNMPAAMVGLPTPAGHQAELNYIYGLVEELSRQLAQNQRALEEVVSGVGKVRGRARSQSLTNDELLN
AAGEELKNQDENIDQLVSILTEALEKAKFSRDANAALLSQYSQVMYTMLKKFHEYKAKHVSDVAAWHHSYRAQLA
EARAENSRLREQIWEMQAHAGKANELVRRFRAEYDKDEKRWERNVNTKAVRQELRFWKRMAMPELPDDDEFW
SDDDDVIDGAEKERQKEIQKLDAEQQAQAAQAVAEEAAANEAAAESSGGLGEMSGSGSGSNGSGLTGVPPEVP
QPPVS

>SMAC_08794 PRO11
MGTNGVHVSFGAGHEMGGVMGNGSGQPPATEYTLQGVMRFLQTEWHRHERDRNAWEIEKQEMRARIANLEGQ
ARRADATQKALKRYVSILEKKVKEQSAALKSGGKEKGVEDEAEAEDNKTKLDRSALIQEKLRPNTKLSGAPNEAEN
VEIGPPDDESARNELKTFLDQCQAEFTYLMVTPANPQPPRESPPLPILDDFREGEALNQQLEQSYQRSQQSQIRD
LLAQQAAAANATNVAAVQSQQQQPQQQQHAVGPRPQTNQPPKGYGELAQGAPMERSSRGQQLHDILKSGETP
DWSASMQSQPVEEPTSALSKAPPMVRGDGREPAVPKSASTTDAWDFNESNFQDPNAMPQPTPQQSFSSRPDT
DAFPSAENLTKSPNRGALSHRRKSSSGMARRRSAEHELSLGSLQQKAEGTMFKFRFGLRGHLDVVRTVIFSGGG
SPGEPEICTAGDDGLVKRFHIPDRHIGQRNADGDLDVAADFTHRGHSGAVLSLASWSPSPNFSTGGRAQGDGWI
FSGGQDATIRVWERGRVDPKATLDGHTDAVWALCVLPTNLGAIFGQTNSHGPPDRILLVSGAADGTVKVWAVSAP
PQLTSPQPGPGRRGPGGRVRGNSMSSGSAFPSSPQPTTASNSPFHHTLVHTIKRANSEASPTCITALSPNGETFA
VSYADAAIIVYDTRSGEEIGTMASLDTYDGTNNTAVNAVVATTVGLDQTQGPGGEDESGGGPTGGGRSMAGSGV
EGTIISGHEDRYIRFFDANSGQCTYNMLAHPGSVSSLSLSPDGRELVSAGHDMSLRFWSLETRSCTQEMTCHRS
MRGEGVCAVVWSQDGRWVVSSGGDGMVKVYAR

>SMAC_00877 SmMOB3
MSLPLSSPRLPSPPPAPEIQIGPKSPAMGPIANRQAQQYEMPTIDLLSHRRIHPGTKAADMAAGPPLVPLHQLDSA
FQLQEHLAALHWHHTAGNTTPITRETAKILATPPAHIDKTVWLYELCRFLITQCNNLIVGFLFDEPPCSAATCPEMRA
SEWQFLCAVHDAPKSCCAIDYCCHTLDWAANTVTNPKYFPSRFFVDTHDKNLALRHLVNIFRRLHRMFAHAWFQH
RSVFWAVEGQTGLYIFFKTVCDHFKTLQHENFQLPPEAEGLESTTTTSGDDDAPQVVIEKPKTHQPGGSVTFAKP
PVIASRGTPIGEPAADDSLMRTNTRRHIKSSPSVGSALTTVPDAEEEDGSSTANTRGKSKVTHRRVPELEPATEEE
METDNIPVIVYHGAAEPQAPHQPTGEAETTEPEDSTSADTAAASPPVEVQKPETEKLESEEVTEEATTSAHGPVQ
EGEHASEDQPTEKSTSDETNEESAEVDHDEVIQPGNEQDKGSESTDVAAHNEAATSAIPALVSESEDSDVDTPAS
SSGSANDDPIGDDQELDKNPTTNTVPDAESEEKPAGEEPTLESNDDGSGEKQNDHHDETKESTSESDSAQSDTN
TAQEEEKEEEKEKEEEKEKEAEKEEEEKGEGKGEKDDKKDKKSDEEATAPVAEQAPKVD

>SMAC_02580 PRO22
MTIMNALWSKATSSSGDSAQKSAKPTNDAVNTTSEALPDLSERPNAPALPARPALQRNQPSAPPPAAPSNPPPPV
PSGSGSGNANNASAQPQDSLSLAQLRRIVAEFPKSEPIAYDYVYSDMGPIEEEVDEWFFYDFWQYVRLNNANHA
FDSAWVKMYGEDETWESVDDDGREKFVRNEVEQLQVSSDKTARVEAIGTLLYIVLGRWTATVKKANLPNLADHKV
KSAATKEQLDAMKAGVKLLAKYGGLPPMWDALRKAFELFWADDGDASQRVQANNEELMELMHLMTILYISLQTT
MDDVEDMAVARKELLALNPNLVHFMLHATAKLRWDDRNILPQAQVGSATFGIPVAANQHRMLMSSQVFLLFWKSL
LLVFGGSKHIAEAKKATAETLSDVKDKEIITASPLDYHVFRQEITSKYPSYIPPQCAIPLEAEQTSILPPLPNHPTRNN
GQNGILPGPPNQSASASILHQPVHIATPAPSPPPSPGVGGKGGKKQNYQTNQNFPFMYPPLDATSNSAGGKGGA
GLQDLLVGRKWEGSDVPASIIEAGELFSTRTRMTRATRQLWEERERFLKFERGWEGADEDLIDELDLSELTLEEKE
ELGLLKESEKKKEKHGHEIDLGPRPVDDDIKRRLEAVEEFYKEALPHLQSLVIVLLKAIVAIASSFVQPPPGQQNPGP
QNNGRAGGGPPQGRGQNNGNNASNDPPSPSDDNVDEARSREIAAKAVTGIMILLLKWLKLSHILKFEYFTQLLLD
SNYLPLVLKLFALHDVQQVVESKTDRIEHSFFYFCASRSGVIPPQGLINPTATDFEDVDVSEDEAAPPAIKRNRSPP
GAEKGGPPDASSQPTQQQQQQQRFENSDVQSRPEVDELGYPVNPLPKEPITDFSRRNFFSLINYLRVMQKICKH
KAHRNLLLVHYKSSNILRKSLKVPQQELRLYTLKLFKNQVPYCGRKWRQSNMRVITAIYLHCRPELRDEWLSGSDV
DAEVEEALPLEQALRSLTHWFNVQRYPERMGAEVTAAMREERDFFTRELEKSDWMGWEGIMAGGGMGMDGGG
GGPEPMMGMQGMQGMPGMPGMGMGMNMGMPGMGIGGMPGMGGMAGIEYAAAAAAAQTEHENSMGWS

Figure S4: Modification sites mapped on the sequences of significantly enriched proteins.
In the SCI1-BioID experiment, the STRIPAK components PRO11, SmMOB3 and PRO22 were significantly enriched. Biotinylated lysine residues are shaded in purple, phosphorylated serine, threonine or tyrosine residues are colored green and oxidations of methionine are shaded blue. No modified peptides of PRO22 were detected with the chosen settings for variable modifications.

References

1. Hollstein LS, Schmitt K, Valerius O, Stahlhut G, Pöggeler S (2022) Establishment of *in vivo* proximity labeling with biotin using TurboID in the filamentous fungus *Sordaria macrospora*. Scientific Reports 12:17727. https://doi.org/10.1038/s41598-022-22545-x
2. Nowrousian M, Stajich JE, Chu M, Engh I, Espagne E, Halliday K, Kamerewerd J, Kempken F, Knab B, Kuo H-C, Osiewacz HD, Pöggeler S, Read ND, Seiler S, Smith KM, Zickler D, Kück U, Freitag M (2010) *De novo* assembly of a 40 Mb eukaryotic genome from short sequence reads: *Sordaria macrospora*, a model organism for fungal morphogenesis. PLOS Genetics 6:e1000891. https://doi.org/10.1371/journal.pgen.1000891
3. Reschka EJ, Nordzieke S, Valerius O, Braus GH, Pöggeler S (2018) A novel STRIPAK complex component mediates hyphal fusion and fruiting-body development in filamentous fungi. Molecular Microbiology 110:513–532. https://doi.org/10.1111/mmi.14106
4. Rappsilber J, Ishihama Y, Mann M (2003) Stop and go extraction tips for matrix-assisted laser desorption/ionization, nanoelectrospray, and LC/MS sample pretreatment in proteomics. Analytical Chemistry 75:663–670. https://doi.org/10.1021/ac026117i
